# Supplementary material for: Genome-wide identification of splicing QTLs in the human brain and their enrichment among schizophrenia-associated loci
Source: Nat Commun. 2017 Feb 27;8:14519. doi: 10.1038/ncomms14519 (PMC5333373; doi:10.1038/ncomms14519)
Supplement: Supplementary Information — Supplementary Figures 1-4, Supplementary Table 1 and Supplementary References [file ncomms14519-s1.pdf]

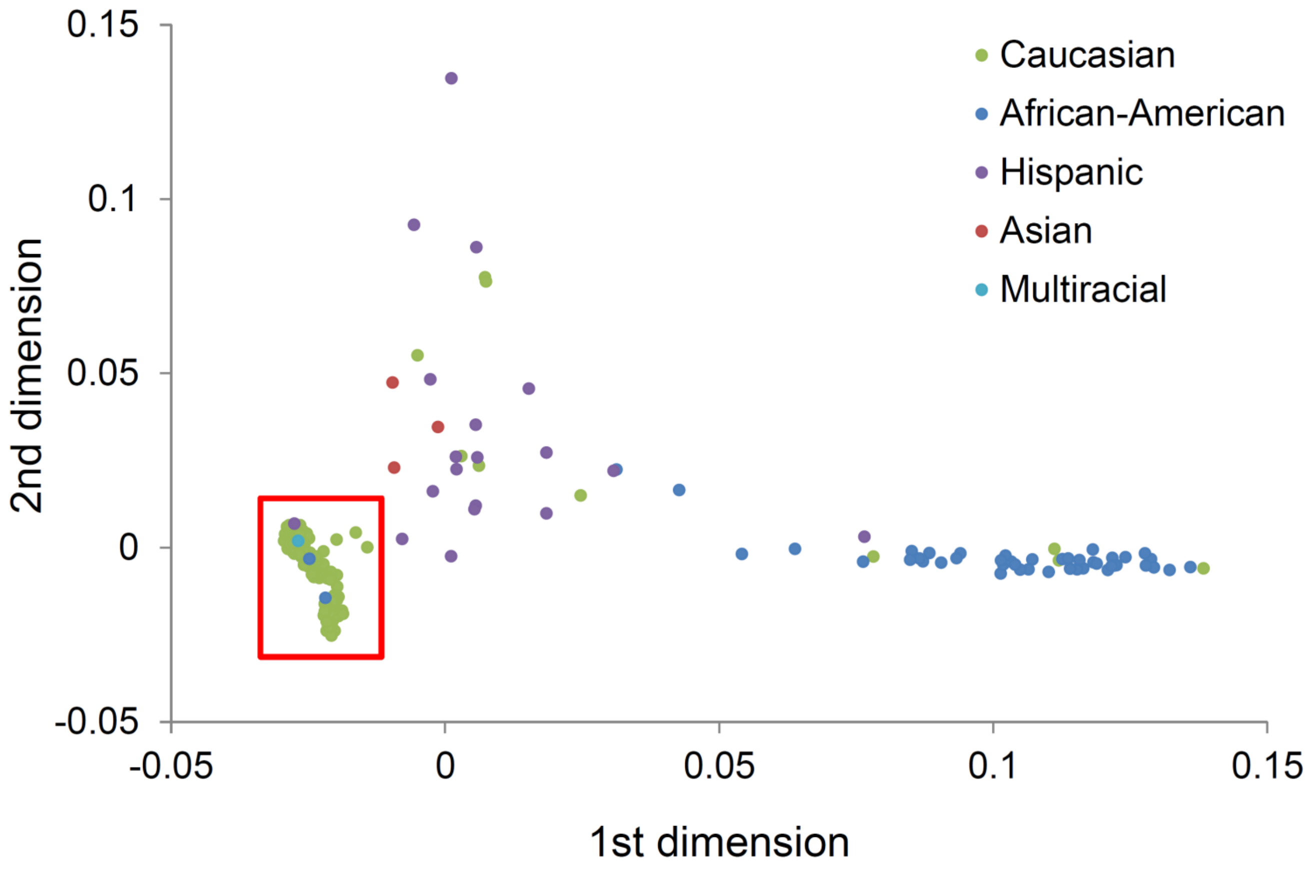

Supplementary Figure 1

Multidimensional Scaling of the Genotyping Data

Multidimensional scaling was performed by using PLINK[1]. Each dot indicates an individual. Dots are color-coded by the ethnicity: green; Caucasian, blue; African-American, purple; Hispanic, red; Asian and light blue; multiracial. We extracted the data of 206 Caucasian individuals in the cluster indicated by the red box and used them in the downstream analyses.

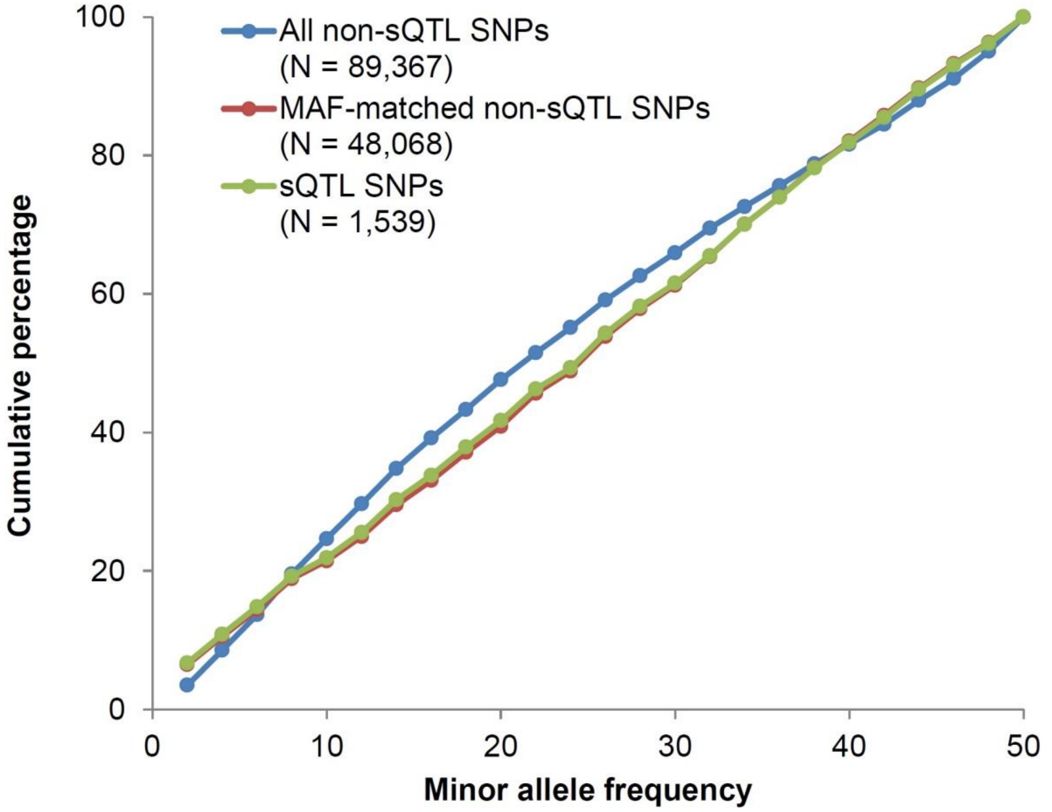

Supplementary Figure 2

Distribution of minor allele frequencies (MAF) in sQTL SNPs, non-sQTL SNPs and MAF-matched non-sQTL SNPs

Cumulative percentages of MAF in sQTL SNPs (green, N = 1,539), non-sQTL SNPs (blue, N = 89,367) and MAF-matched non-sQTL SNPs (red, N = 48,068) were plotted. To generate a set of MAF-matched non-sQTL SNPs, we stratified non-sQTL SNPs into 2% MAF bins and extracted maximum number of SNPs with distribution of MAF matched to sQTL SNPs.

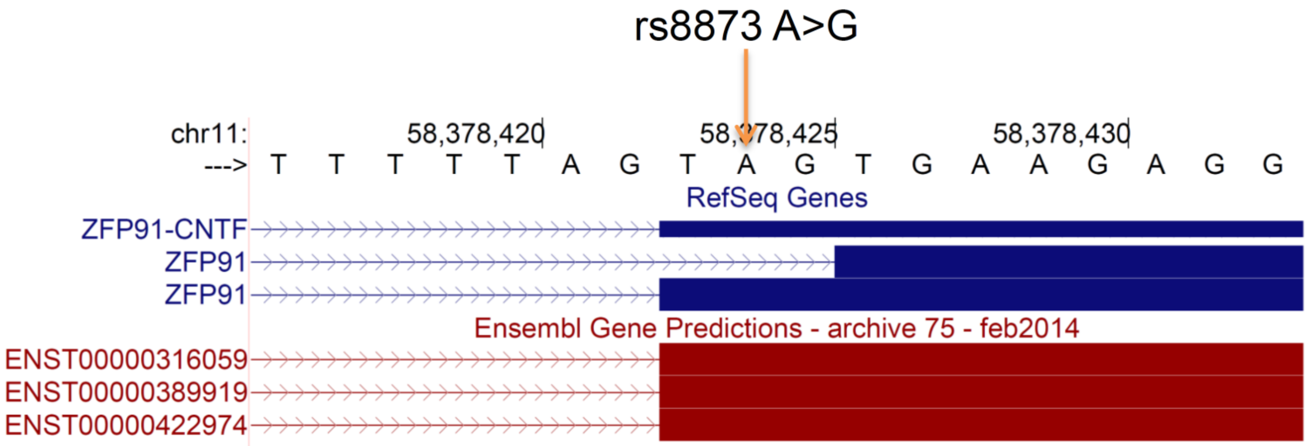

Supplementary Figure 3

A canonical splice site variant without detectable alternative splicing event

The rs8873 variant (chr11: 58,378,424, orange arrow) was classified as a splice site variant according to the annotation based on SnpEff, whereas this SNP is located at a canonical splice site that is only found in the RefSeq Genes track and not in the Ensembl Gene Predictions track of the UCSC Genome Browser (https://genome.ucsc.edu/). By manually inspecting the data of AS at this site, we found that alternative usage of splice site at chr11: 58,378,426 was not detected in our analysis.

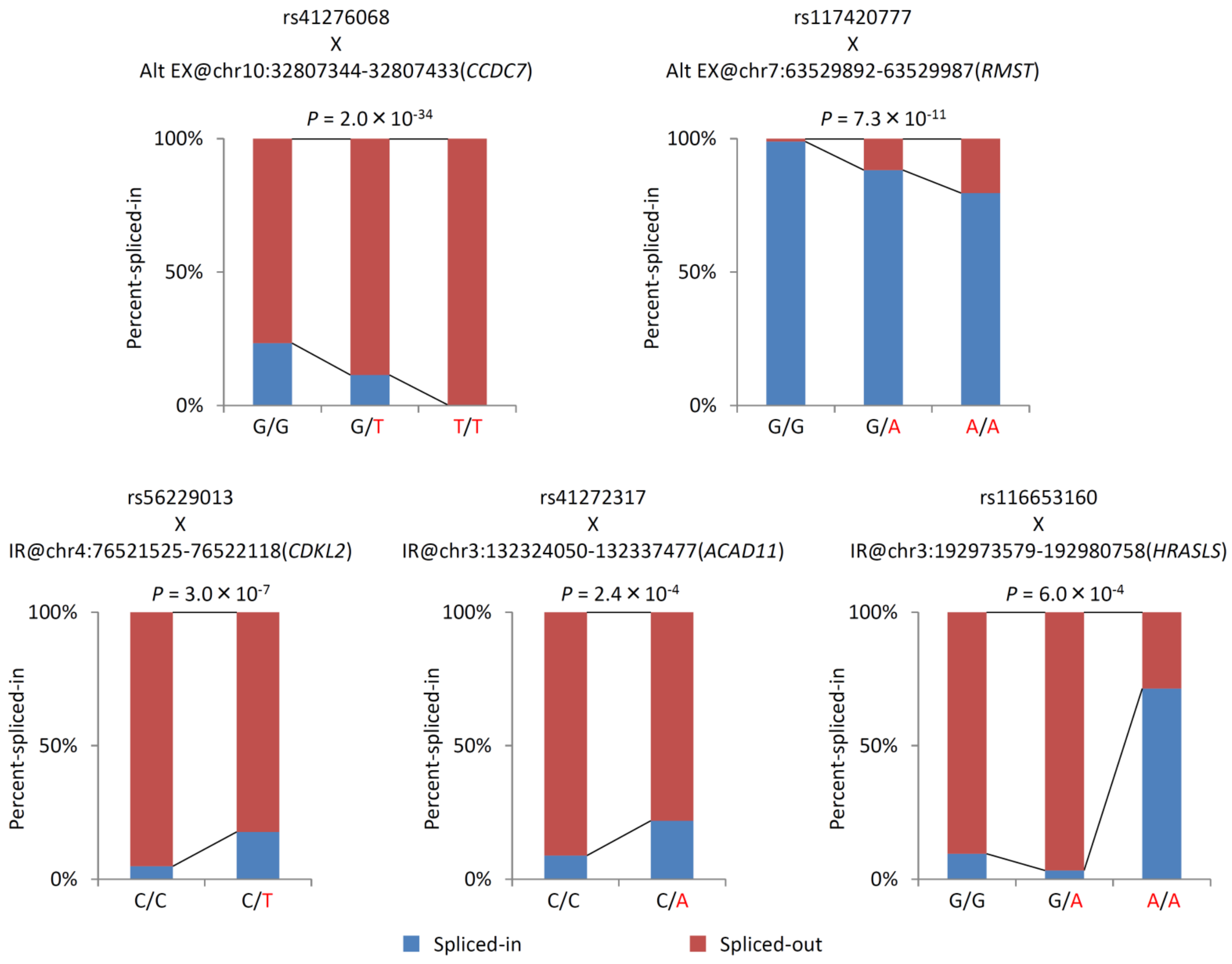

Supplementary Figure 4

Canonical splice site sQTL SNPs associated with AS of the adjacent region

Besides sQTL SNPs shown in **Fig. 2**, which are associated with known AS events (annotated by Ensembl Gene Predictions), we identified five sQTL SNPs significantly associated with an AS of the adjacent region that has not been annotated by Ensembl. For these sQTL SNPs (rs41276068, rs117420777, rs56229013, rs41272317 and rs116653160), we plotted average percent-spliced-in (PSI) of the associated AS in each genotype as stacked bars (blue: spliced-in and red: spliced-out). Double-corrected *P* values (see **Methods**) are indicated above the bars. The variant alleles disrupting a canonical splice site are indicated by red letters. For rs56229013 and rs41272317, there was no individual homozygous for the variant allele.

Supplementary Table 1

Summary Statistics of the Samples/Individuals Included in This Study

Variables	Whole dataset (N=206)	Mt. Sinai (N=108)	Pennsylvania (N=25)	Pittsburg (N=73)
Age of death (years, average ± SD)[a]	64.6 ± 19.4	75.1 ± 16.4	68.5 ± 16.1	49.5 ± 13.6
Post-mortem interval (hours, average ± SD)	14.5 ± 7.8	11.9 ± 8.0	13.5 ± 7.3	19.2 ± 5.2
pH (average ± SD)	6.6 ± 0.3	6.5 ± 0.3	6.4 ± 0.3	6.7 ± 0.2
RNA integrity number (average ± SD)	7.8 ± 0.9	7.4 ± 0.9	7.5 ± 0.7	8.5 ± 0.4
Mapped number of reads (millions, average ± SD)	78.6 ± 20.6	76.9 ± 17.7	87.1 ± 19.9	78.3 ± 24.2
Total number of reads (millions, average ± SD)	87.4 ±24.3	85.5 ± 18.5	95.1 ± 21.3	87.7 ± 31.4
Percent aligned (average ± SD)	90.0 ±4.6	89.8 ± 5.1	91.5 ± 2.9	89.9 ± 4.3
Gender (male: female)	119: 87	54: 54	13: 12	52: 21

[a]Age of death ≥ 90 was considered as 90 due to limited information in the original data source.

Supplementary Reference

1. Purcell S, Neale B, Todd-Brown K, Thomas L, Ferreira MA, Bender D *et al.* PLINK: a tool set for whole-genome association and population-based linkage analyses. *Am J Hum Genet* 2007; **81**(3)**:** 559-575.
